# Self-Reported Musculoskeletal Disorders and Quality of Life in Supermarket Cashiers

**DOI:** 10.3390/ijerph17249256

**Published:** 2020-12-10

**Authors:** Fahad Saad Algarni, Hatem Askar Alkhaldi, Hamayun Zafar, Shaji John Kachanathu, Abdullah M. Al-Shenqiti, Abdulrahman Mohammed Altowaijri

**Affiliations:** 1 Department of Rehabilitation Sciences, College of Applied Medical Sciences, King Saud University, Riyadh 11433, Saudi Arabia; hzafar@KSU.EDU.SA (H.Z.); skachanathu@KSU.EDU.SA (S.J.K.); 2 Department of Rehabilitation Medicine, King Saud University Medical City, King Saud University, Riyadh 11461, Saudi Arabia; halkhaldi@ksu.edu.sa (H.A.A.); abaltowaijri@KSU.EDU.SA (A.M.A.); 3 Faculty of Medical Rehabilitation Sciences, Taibah University, Medina 42353, Saudi Arabia; alshenqitirehab@hotmail.com

**Keywords:** supermarket cashiers, repetitive work, musculoskeletal disorders, quality of life, occupational injuries, work-related disabilities

## Abstract

Supermarket cashiers face a significant amount of stress, including time constraints, mental pressure, and physical demands that require repetitive movements. The job description of a supermarket cashier involves work-related risk factors that may lead to musculoskeletal disorder (MSD) symptoms. This study aimed to investigate supermarket cashiers to determine the prevalence of MSD symptoms and their quality of life. Data were collected from a convenience sample of supermarket cashiers working in Saudi Arabia. Information included direct questions on pain in the previous 12 months, demographic data, and health- or occupation-related factors. Moreover, data was collected based on the 36-item short form survey (SF-36), and descriptive statistics were computed. A total of 193 supermarket cashiers participated in this study. The sample included 140 men (72.5%) and 53 women (27.5%), with a mean age of 27.2 ± 6.4 years. The majority of the participants (90%) had MSD symptoms in at least one body region, with the neck (66.84%) and lower back (65.80%) constituting the most prominent regions. The mean SF-36 scores were higher in participants without pain compared to participants with pain in all domains, except for the physical functioning domain. The high prevalence of MSD symptoms among young cashiers suggest the need for additional investigations to determine the risk factors of these disorders. Additionally, this study recommends preventive procedures to reduce the prevalence of MSD symptoms among cashiers.

## 1. Introduction

Job performance of certain professions requires specific postures and continuous repetitive movements often conducted with forceful excretions that may lead to musculoskeletal disorder (MSD) symptoms [1,2]. The job of supermarket cashiers is among these professions. MSD symptoms (i.e., pain) have a detrimental effect on employees, potentially leading to incapacity and inability to continue working.

Individuals working as supermarket cashiers lack the need for a specific education level, making this job suitable for most individuals. Despite the apparently simple nature of the cashier’s job, this position entails several challenges. Supermarket cashiers spend long hours in relatively static postures within congested areas. Additionally, they handle grocery items of various sizes and swipe barcodes through a laser scanner. Cashiers repeat this task several times, sometimes processing 500 to 1000 items per hour [3]. Moreover, supermarket cashiers face substantial stress, including time constraints, mental pressure, and physical demands, especially with repetitive movements [4]. Therefore, the job description of a supermarket cashier entails job-related risk factors that cause MSD symptoms.

Many studies have been conducted on supermarket cashiers in different countries [5,6,7,8], reporting a widespread prevalence of MSD symptoms. These results demonstrated that approximately 70% of cashiers have shoulder and neck pain [7], while 47% of these employees have wrist or hand pain [6]. Cashiers have more stress load in their upper limbs than in other body parts [5], thus putting cashiers at a high risk for developing MSD symptoms in the upper extremities [9]. Moreover, cashiers incur neuromuscular fatigue in their neck, shoulders, and lower back muscles [10].

Relatively few studies have previously investigated MSD symptoms in cashiers. To date, no existing study has been conducted on supermarket cashiers in Saudi Arabia (SA). Thus, data from previous studies originate from other cultures, demonstrating the need to extend research to SA. Furthermore, the majority of previous studies involved female cashiers [11,12,13,14], which necessitates the requirement to investigate male cashiers. To date, the quality of life of cashiers have not been investigated. Accordingly, the present study focuses on the possible impact of the MSD symptoms on the lives of male and female supermarket cashiers working in SA. Moreover, this study aimed to determine the prevalence of MSD symptoms in various body regions, including the neck, shoulders, upper limbs, lower back, and lower limbs. Finally, this study compared the quality of life of cashiers with and without MSD symptoms.

## 2. Materials and Methods

This study employed a descriptive cross-sectional study involving supermarket cashiers of both sexes between ages 18 and 60 years in SA. Cashiers with malignant tumors or previous musculoskeletal injuries, such as fractures, dislocations, or previous surgeries in the spine and limbs, were excluded. Ethical approval was obtained from the Research Ethics Committee of King Saud University, Riyadh, while informed consent was obtained from each participant.

The study used a non-probability sampling technique to recruit participants from different supermarkets in all cities. Subsequently, a website questionnaire collected the required data by piloting a group of cashiers similar to the targeted sample. Participants sent the website link to their friends and colleagues, while social media influencers on platforms such as Twitter, Snapchat, and Instagram sent the link to supermarket cashiers across SA. In conducting the study, single proportion calculation was performed to estimate the sample size. The estimated sample size was 180 [6].

The collected data comprised direct questions related to demographics, health status, social habits, and occupational factors. The remaining information showed the presence of MSD symptoms in the past 12 months through direct questions and instruments in the Arabic language. These questions determined the presence of pain in several areas, including the neck, shoulders, or upper back; lower back; elbow, hand, or wrist; hip, thigh, or knee; and ankle or feet, which are similar areas investigated in the Nordic questionnaire. After indicating the presence of pain in any of these areas, cashiers identified the intensity of pain using the numeric rating scale [15]. Furthermore, participants responded to the 36-item short form survey (SF36), which was previously translated and adapted to the Arabic language [16]. Moreover, participants indicated the presence of MSD symptoms before their employment as a cashier, belief that such MSD symptoms were related to their occupation, and intensification of these MSD symptoms with their cashier position.

Data analysis was performed using the Statistical Package for the Social Sciences (SPSS 22; IBM Corp., New York, NY, USA), which computed descriptive statistics for a set of variables or characteristics. All variables underwent classification as either continuous variables, expressed as mean ± standard deviation, or categorical variables, expressed as percentages. The Mann–Whitney U test was used to determine the existence of a significant difference between cashiers with and without MSD symptoms, using a *p*-value of 0.05.

## 3. Results

A total of 213 supermarket cashiers participated in this study. From these, 20 participants failed to meet the inclusion criteria, leaving 193 supermarket cashiers to provide the required data. Among this sample, there were 140 men and 53 women, with a mean age of 27.2 (±6.4) years. A total of 162 (84%) cashiers lacked any history of chronic diseases or medical conditions, while 104 (54%) cashiers refrained from practicing any kind of exercise or sport activities. Table 1 presents all demographic and health-related characteristics.

A total of 87 (45%) cashiers reported up to one year of work experience, while 81 (42%) had work experience of one to four years. Moreover, 163 (85%) cashiers had full-time work, while 109 (57%) cashiers worked for >8 h per day. Among all participants, 131 (68%) felt satisfied with their cashier jobs. Furthermore, 152 (79%) cashiers performed solely cashier-related tasks within the supermarket. More than half of the cashiers (108 or 56%) reported that their supervisors failed to provide them with any safety training for avoiding work-related musculoskeletal disorders (WMSDs). The data showed that, on average, the cashiers stood on their feet for approximately 5.7 h (±2.41). Finally, 121 (63%) participants mentioned using comfortable shoes on the job, with only 7 cashiers (3%) reporting the availability of cushions under their feet at work.

This study investigated other additional tasks that cashiers performed during their duties. A total of 83 (43%) cashiers unloaded groceries from shopping trollies for scanning, while 157 (81%) cashiers packed grocery items in bags. Furthermore, 149 (77%) of cashiers assumed awkward positions to reach items placed in the shopping trolley. Specifically, Table 2 presents all occupational-related characteristics.

The questionnaire indicated that 174 (90%) participants had musculoskeletal pain at least in one body area in the past 12 months. Cashiers reported MSD symptoms in all body areas with varying prevalence. Particularly, the neck and lower back regions constituted the two most affected areas among participants, with 129 (67%) having neck pain and 127 (66%) having back pain. Table 3 shows the prevalence of MSD symptoms in different body areas.

As show in Table 4, the pain intensity, Numeric Pain Rating Scale, (NPRS) in all body regions ranged from 5.1 to 6.0. The highest pain level was noted in the ankle/foot, at 6.0 (±2.6), while the least pain level was noted in the neck, at 5.1 (±2.2), and the hand/wrist, at 5.1 (±2.5). Moreover, participants reported that their cashier work aggravated previously existing pain, with the values of aggravation varying from one body area to another (Table 4).

More comparisons were done (see Appendix A) in order to investigate whether there was a difference of location of the MSD symptoms (body regions) across different groups and key variables (e.g., age, gender, marital status, dominant hand, educational level, smoking status, job experience, etc.). Cashiers with neck MSD symptoms were significantly different than cashiers without across the following variables: marital status, using medications, proper training prepared to avoid WMSDs, and job satisfaction. Regarding upper back MSD symptoms, cashiers with symptoms were significantly different than those without across educational level, job satisfaction, and packing groceries. Cashiers who reported lower back MSD symptoms were significantly different than those without across the following variables: dominant hand, using medications, proper training prepared to avoid WMSDs, number of breaks, and job satisfaction.

Significant differences were found among the two groups (cashiers with and without MSD symptoms of upper limb). Shoulder symptoms were significantly different between the two groups of cashiers across number of times practicing sport per week, using medications, and job satisfaction. Regarding elbow joint, the two compared groups had significant differences across the following variables: using medications and job satisfaction. Regarding hand and wrist joint, the two groups of cashiers had significant differences across the following variables: using medications, proper training prepared to avoid WMSDs, and number of breaks.

The MSD symptoms in the lower limb also had significant differences across some variables between the two groups of cashiers. Regarding the hip joint, the two groups were significantly different across dominant hand, and using medications. The knee symptoms had significant differences between the two groups across some variables (job experience, number of breaks, work type, and job satisfaction). Finally, MSD symptoms of the ankle had significant differences between the two groups with respect to the following variables: smoking, using medications, proper training prepared to avoid WMSDs, number of breaks, and packing groceries.

Regardless of whether they had pain, all participants completed the SF-36. As shown in Table 5, all SF-36 domains significantly differed between individuals with and without MSD symptoms, except for the physical functioning (PF) domain.

## 4. Discussion

This study found that 90% of supermarket cashiers in SA have MSD symptoms in the past 12 months. These cashiers reported that the neck and lower back regions constituted the two most affected areas, while the hip/thigh area and elbow represented the least affected regions. Compared to cashiers without MSD symptoms, all cashiers with MSD symptoms had decreased quality of life. The results of this study are in line with the previous study from Estonia, which reported that approximately 87% of supermarket cashiers had pain in at least one body region in the last six months [6].

These results concur with previous studies [5,6,8,11,12], indicating the prevalence of MSD symptoms in supermarket cashiers. However, while most existing literature investigated female cashiers [5,6,11,12], the majority of participants in this study (140 or 72.5%) were male. Additionally, the study revealed significant differences in the musculoskeletal system between men and women [17]. Compared to previous studies, which reported mean cashier ages ranging from 33 to 40 years [6,12], this study found a mean age of 27.2 years (±6.4 years) among cashiers in SA. Moreover, most cashiers in this study reported work experience of less than one year, which is less than that in previous studies [7,12]. Particularly, the Saudization program, which requires all supermarkets in SA to hire Saudi staff, may account for this difference and the fact that older SA workers may lack an interest in this position due to the low salary.

Furthermore, most cashiers in this study have a body mass index (BMI) that qualifies as overweight. Increased BMI represents a risk factor for work-related MSD symptoms [18]. Unfortunately, more than half (54%) of cashiers reported sedentary behavior. Since the aim of this study did not include the examination of the relationship between high BMI and MSD symptoms prevalence, future investigations should seek to determine the potential association between these two factors. However, one study found evidence that physical activity may decrease the prevalence of MSD symptoms in the working-age population [19].

Moreover, the study found that 68% of participants reported satisfaction with their job. Despite the high job satisfaction rate, the prevalence of MSD symptoms remained high among participants. This relationship demonstrates inconsistency with a previous systemic review, which reported that low job satisfaction constitutes a risk factor for back pain [20].

The current study did not evaluate the relationship between the reported MSD symptoms and cashiers’ jobs. The present results show that the majority of cashiers believes that the nature of their job may have somewhat contributed to their MSD symptoms. However, this apparent relationship was based on the cashiers’ opinion, which lacks objective evidence or validated methods for examining the causal relationship between MSD symptoms and work.

The current study found that the highest prevalence of MSD symptoms in a 12-month period involved the neck and lower back areas, with 67% of respondents having neck pain and 66% of participants having low back pain (LBP). The present study demonstrated a higher prevalence of neck pain than a previous study, with results reporting that 54% of participants had neck pain in the past 6 months [6]. The possible justification for the high prevalence of neck pain in the present study may be the cashier’s desire to work in the standing position, which requires more range of motion (ROM) of the neck [13]. However, the combined prevalence of shoulder and neck pain among supermarket cashiers reached 70% in other studies [7]. Additionally, previous evidence found that cashiers had the same degree of neck pain within the period of seven days [14]. Neck pain remains common in jobs characterized by repetitive or monotonous motions [21], such as cashier jobs. Several factors influence neck postures and muscle and ligament alignment. Specifically, neck muscle activity level increases with the spine in a flexion position [22], such as sitting. Even in the standing position, cashiers require more neck flexion [13]. Therefore, supermarket cashiers have a relatively high degree of MSD symptoms from the neck.

According to the European Agency for Safety and Health at Work, LBP is one of the main causes of disability and sick leave [23]. Back pain, in particular, comprises one of the most common problems among supermarket cashiers [9]. The process of sitting for long periods of time increases the likelihood of LBP by 1.5 times. In fact, studies have found an association between prolonged standing in the same position and LBP [24]. The current study showed similar results, as 66% of supermarket cashiers reported LBP, which concurred with the results of Sirge et al. (2014), reporting an LBP prevalence of 67% among cashiers [6]. In cashier work, the lower back is subject to injury, as cashiers usually perform trunk forward flexion, lateral leaning, and rotation movements [5]. The combination of such movements comprises the main factors associated with the development of back problems [25]. Moreover, the need to handle heavy items requires more ROM in the trunk, which may result in the high prevalence of lower back disorders among cashiers [26].

In a cashier job, the upper limbs bear more stress load than other body parts [5]. Due to the nature of their job, cashiers have a high risk of developing MSD symptoms in the upper extremities [9]. Approximately 55% of current participants reported shoulder pain, demonstrating consistency with previous studies revealing the prevalence of shoulder pain among supermarket cashiers as 51% [27] and 43% [6]. Compared to a standing position, the requirement to work in a sitting position requires more shoulder muscle activation [4] and ROM [28]. Additionally, cashiers experience the three factors present in shoulder-related disorders: presence of flexion, repetitive arm tasks, and high-speed functionality [4]. In the current study, 40% of participants reported hand or wrist pain. Balogh et al. (2016) found that cashiers demonstrated a 34% prevalence of hand symptoms [14]. This prevalence was noted in cashiers because of the nature of their work, which requires repetitive movements of the wrist joint [29].

Finally, the lowest prevalence of MSD symptoms in the present study involved the elbow joint. This percentage concurred with the results of Sirge et al. (2014), who found that 19% of cashiers had elbow pain [6]. This finding may result from the fact that 71% of participants preferred working in a standing position, which may require less ROM in the elbow joint [28].

Since previous studies neglected to address the prevalence of MSD symptoms in the lower limbs, the current study investigated this region to fulfill previous research gaps. Results found that approximately 37% of participants in the present study reported knee pain. This finding shows consistency with a previous study, which reported that 39% of female cashiers had knee pain in the previous six months [6]. Anton and Weeks (2016) evaluated all supermarket workers, including cashiers, and found a higher prevalence of foot pain among workers spending most of their time in a standing position than among those who spend most of their time walking [30]. The current study found a similar prevalence of MSD symptoms in the ankle/foot. Finally, this study reported that 33% of cashiers had MSD symptoms in the hip/thigh. Due to the lack of previous studies investigating the prevalence of MSD symptoms in the lower limbs of cashiers, this study lacks an adequate comparison. Since cashiers spend a long time in a standing position, which distributes body weight to the lower limbs, these employees had constant contraction of the leg muscles [31], which can cause lower body MSD symptoms.

According to the body regions (see Appendix A), significant differences were found between cashiers with and without MSD symptoms across several variables. There are three variables that are more likely to make a difference between cahiers with and without MSD symptoms. These variables are using medications, job satisfaction, and proper training prepared to avoid WMSDs. However, this issue was not under the scope of the current paper. Future studies should make more investigations for such differences of the location of the musculoskeletal symptoms across these characteristics/variables.

This study represents the first work to evaluate the health-related quality of life in supermarket cashiers, as previous studies have neglected to address quality of life among these employees. Moreover, no studies have yielded data regarding the health-related quality of life for the general Saudi population. Nevertheless, this study compared the quality of life of participants with MSD symptoms to those without MSD symptoms (Table 5). With the exception of PF, all domains of SF-36 scores were significantly higher in participants without MSD symptoms than those with MSD symptoms. Therefore, healthy participants have a higher quality of life than those with MSD symptoms. The finding that MSD symptoms affect the quality of life of supermarket cashiers concurs with the results of a previous study conducted on 3664 participants with equal numbers of men and women aged ≥25 years. The results of that study showed that, compared to participants with MSD symptoms, those without MSD symptoms have significantly higher SF-36 scores in all domains [32].

Compared to cashiers without MSD symptoms, those with MSD symptoms had more difficulties with social activities due to emotional problems. In addition, cashiers with MSD symptoms had lower vitality than their non-MSD symptoms counterparts. Similarly, those with MSD symptoms had more feelings of depression or anxiety than those without MSD symptoms. Individuals with MSD symptoms also exhibited worse social functioning than those without MSD symptoms. Moreover, cashiers with MSD symptoms had more pain or limitations than those without MSD symptoms. Finally, cashiers with MSD symptoms had more negative perceptions of their general health compared to those without MSD symptoms. Since the present study constitutes the first work to investigate the quality of life among cashiers, future studies are recommended to achieve more insight into this issue.

This study used a non-probability sampling technique. However, the sampling attempted to reduce selection bias by contacting various supermarkets from different companies and cities. Moreover, this study recruited solely cashiers in active service, thus neglecting those who had left the workforce due to MSD symptoms or other reasons. In the current study, only 19 cashiers were found to be without MSD symptoms which may indicate the possibility of confounding factors. Moreover, future studies may consider pain severity to determine case status, with appropriate cut-off points. Lastly, this study employed a self-reported questionnaire investigating MSD symptoms and their effects on daily activities. The use of a survey may have overestimated or underestimated previous symptoms and their effects on activities.

## 5. Conclusions

Supermarket cashiers have a high risk of developing MSD symptoms due to the nature of their work. This study found a high prevalence of MSD symptoms, especially in the neck and lower back, in supermarket cashiers working in SA. The presence of MSD symptoms influenced functional levels and quality of life of cashiers. This study recommends proper training and work organization as methods of preventing or minimizing MSD symptoms among supermarket cashiers in SA.

## Figures and Tables

**Table 1:** Summary statistics of demographic and health-related data.

Variables	Categories	N (%) or *x* ± SD
**Age (years)**		27 ± 6
**Height (cm)**		165 ± 11
**Weight (kg)**		74 ± 21
**BMI (kg/m^2^)**		27 ± 8
**Sex**	Male	140 (73)
	Female	53 (28)
**Marital status**	Single	123 (64)
	Married	55 (29)
	Others	15 (8)
**Person taking care of children during a work shift**	Baby sitter	7 (14)
	Grandfather or grandmother	8 (15)
	Wife	27 (52)
	Husband	7 (14)
**Nationality**	Saudi	184 (95)
	Non-Saudi	9 (5)
**Dominant hand**	Right	170 (88)
	Left	23 (12)
**Educational level**	Intermediate school or lower	23 (12)
	High school	123 (64)
	Diploma/Bachelor	47 (24)
**City of work**	Inside Riyadh	157 (81)
	Outside Riyadh	36 (19)
**Smoking status**	Smoker	66 (34)
	Non-smoker	117 (61)
	Ex-smoker	10 (5)
**Practice sports times/week**	No practice of sports	104 (54)
	1–3 times/week	68 (35)
	4 times or more/week	21 (11)
**History of disease (chronic disease or medical conditions)**	Yes	31 (16)
	No	162 (84)
**Us** **ing medications (any medication)**	Yes	36 (19)
	No	157 (81)

**Table 2:** Summary statistics of occupation-related data.

Variables	Categories	N (%) or *x* ± SD
**Working days per week (days)**		5 ± 0.7
**Number of breaks during a work shift, mean (SD)**		1.7 ± 1.2
**Number of sitting hours, mean (SD)**		2.7 ± 2.2
**Number of standing hours, mean (SD)**		5.7 ± 2.4
**Job experience (years)**	One year	87 (45)
	>one year–4 years	81 (42)
	≥5 years	25 (13)
**Work type**	Full-time	163 (85)
	Part-time	30 (16)
**Job satisfaction**	Satisfied	131 (68)
	Not satisfied	62 (32)
**Used a laser scanner (barcode scanner)**	Yes	176 (91)
	No	17 (9)
**Used a bioptic scanner (vertical and horizontal), making work easier**	Yes	168 (87)
	No	25 (13)
**Direction of receiving groceries coming through the conveyor belt**	From the right side	148 (77)
	From the left side	45 (23)
**Reached for items placed in the shopping trolley (awkward position)**	Yes	149 (77)
	No	44 (23)
**Unloaded groceries from carts for scanning**	Yes	83 (43)
	No	110 (57)
**Packed groceries items into bags after scanning**	Yes	157 (81)
	No	36 (19)
**Preferred position for working**	Standing	137 (71)
	Sitting	56 (29)
**Practicing for other tasks in the supermarket besides working as a cashier (job rotation)**	Yes	41 (21)
	No	152 (79)
**Had another job outside the supermarket**	Yes	8 (4)
	No	185 (96)
**Received proper training to avoid WMSDs before starting work as a supermarket cashier**	Yes	85 (44)
	No	108 (56)
**Had a chair to sit on at a workstation**	Yes	161 (83)
	No	32 (17)
**Used comfortable shoes while working**	Yes	121 (63)
	No	72 (37)
**Presence of a foot rest cushion under the feet while standing at work**	Yes	7 (4)
	No	186 (96)
**Number of workers at the same supermarket where the cashier works**	>50 workers	79 (41)
	<50 workers	114 (59)
**Working hours per day**	>8 h	109 (57)
	≤8 h	84 (44)

**Table 3:** Prevalence of musculoskeletal disorder (MSD) symptoms with respect to body region in the previous 12 months.

MSD Symptoms	N (%)
**At least one body area**	174 (90)
**Neck**	129 (67)
**Shoulder joint**	107 (55)
**Upper back area**	111 (58)
**Lower back area**	127 (66)
**Elbow joint**	47 (24)
**Hand/wrist joint**	78 (40)
**Hip joint/thigh**	63 (33)
**Knee joint**	71 (37)
**Ankle joint/foot**	75 (39)

**Table 4:** Details of prevalence of MSD symptoms in the body regions.

Body Region	Yes/No	Neck	Shoulder	Upper Back Area	Lower Back Area	Elbow	Hand/Wrist	Hip/Thigh	Knee	Ankle/Foot
		N (%) or *x* ± SD	N (%) or *x* ± SD	N (%) or *x* ± SD	N (%) or *x* ± SD	N (%) or *x* ± SD	N (%) or *x* ± SD	N (%) or *x* ± SD	N (%) or *x* ± SD	N (%) or *x* ± SD
Prevalence of pain in the previous 12 months		(67) 129	(55) 107	(58) 111	(66) 127	(24) 47	(40) 78	(33) 63	(37) 71	(39) 75
Pain intensity: NPRS, mean (SD)		±2.2 5.1	±2.46 5.4	±2.4 5.6	±2.6 5.9	±2.5 5.3	±2.5 5.1	±2.5 5.7	±2.5 5.4	±2.6 6
Participants’ beliefs about the correlation between pain and nature of their work.	**Yes**	(81) 104	(89) 95	(93) 103	(86) 109	(89) 42	(89) 69	(89) 56	(80) 57	(96) 72
	**No**	(19) 25	(11) 12	−7.2 8	(14) 18	(11) 5	(12) 9	(11) 7	(20) 14	4 3
Pain present before working as supermarket cashier	**Yes**	(22) 28	(16) 17	(18) 20	(32) 41	(11) 5	(19) 15	(18) 11	(24) 17	(19) 14
	**No**	(78) 101	(84) 90	(82) 91	(68) 86	(89) 42	(81) 63	(83) 52	(76) 54	(81) 61
Pain aggravation after working as a cashier. (For those who reported experiencing pain prior to working as a supermarket cashier).	**Yes**	(50) 14	(77) 13	(80) 16	(68) 28	(60) 3	(60) 9	(82) 9	(71) 12	(79) 11
	**No**	(50) 14	(24) 4	(20) 4	(32) 13	(40) 2	(40) 6	(18) 2	(29) 5	(21) 3

**Table 5:** Summary of 36-item short form (SF-36) scores.

Scales	Mean (SD) for Cashiers without MSD Symptoms	Mean (SD) for Cashiers with MSD Symptoms	*p*-Value
	Mean ± SD	Mean ± SD	
Physical functioning (PF)	66.6 ± 33.8	67.8 ± 26	NS
Role limitations due to physical health (RP)	80.3 ± 31.8	60.3 ± 38	*
Role limitations due to emotional problems (RE)	89.5 ± 25	54.2 ± 41.1	***
Energy/fatigue (VT)	75.3 ± 17.8	56 ± 21	***
Emotional well-being (MH)	81.7 ± 16.3	62.6 ± 21	***
Social functioning (SF)	85.5 ± 18.8	61.3 ± 27.3	***
Pain (BP)	87.4 ± 18.6	65.2 ± 25.5	***
General health (GH)	75.8 ± 10.6	62.4 ± 15.2	***

The final score of the SF-36 ranges from 0 to 100, with higher scores meaning higher levels of function and/or better health. NS, not significant, * *p* < 0.05, *p* < 0.01, *** *p* < 0.001. *p*-value was calculated using Mann–Whitney U test.

## Appendix A

**Table A1:** Spine.

		Neck					Upper Back					Lower Back				
		MSD Symptoms														
Variables		Yes		No		*p*	Yes		No		*p*	Yes		No		*p*
		N	%	N	%		N	%	N	%		N	%	N	%	
Gender	Male	94.0	67.1	46.0	32.9	0.88	75.0	53.6	65.0	46.4	0.07	92.0	65.7	48.0	34.3	0.97
	Female	35.0	66.0	18.0	34.0		36.0	67.9	17.0	32.1		35.0	66.0	18.0	34.0	
Marital status	Single	86.0	69.9	37.0	30.1	0.02	70.0	56.9	53.0	43.1	0.88	84.0	68.3	39.0	31.7	0.09
	Married	38.0	69.1	17.0	30.9		33.0	60.0	22.0	40.0		37.0	67.3	18.0	32.7	
	OTHER	5.0	33.3	10.0	66.7		8.0	53.3	7.0	46.7		6.0	40.0	9.0	60.0	
Dominant hand	right	115.0	67.6	55.0	32.4	0.52	99.0	58.2	71.0	41.8	0.58	118.0	69.4	52.0	30.6	0.00
	Left	14.0	60.9	9.0	39.1		12.0	52.2	11.0	47.8		9.0	39.1	14.0	60.9	
Educational level	Intermediate or less	15.0	65.2	8.0	34.8	0.06	10.0	43.5	13.0	56.5	0.01	14.0	60.9	9.0	39.1	0.54
	Secondary	76.0	61.8	47.0	38.2		65.0	52.8	58.0	47.2		79.0	64.2	44.0	35.8	
	Diploma	38.0	80.9	9.0	19.1		36.0	76.6	11.0	23.4		34.0	72.3	13.0	27.7	
Smoking status	Smoker	46.0	69.7	20.0	30.3	0.19	41.0	62.1	25.0	37.9	0.61	46.0	69.7	20.0	30.3	0.70
	Non smoker	74.0	63.2	43.0	36.8		65.0	55.6	52.0	44.4		75.0	64.1	42.0	35.9	
	X—smoker	9.0	90.0	1.0	10.0		5.0	50.0	5.0	50.0		6.0	60.0	4.0	40.0	
Practice sports times/week	No practice of sports	73.0	70.2	31.0	29.8	0.46	56.0	53.8	48.0	46.2	0.48	73.0	70.2	31.0	29.8	0.25
	1–3 times/week	44.0	64.7	24.0	35.3		43.0	63.2	25.0	36.8		43.0	63.2	25.0	36.8	
	4 times or more/week	12.0	57.1	9.0	42.9		12.0	57.1	9.0	42.9		11.0	52.4	10.0	47.6	
Using medications (any medication)	Yes	31.0	86.1	5.0	13.9	0.01	25.0	69.4	11.0	30.6	0.11	31.0	86.1	5.0	13.9	0.00
	No	98.0	62.4	59.0	37.6		86.0	54.8	71.0	45.2		96.0	61.1	61.0	38.9	
Job experience (years)	≤ one year	52.0	59.8	35.0	40.2	0.10	46.0	52.9	41.0	47.1	0.41	58.0	66.7	29.0	33.3	0.97
	2–4 year	61.0	75.3	20.0	24.7		51.0	63.0	30.0	37.0		53.0	65.4	28.0	34.6	
	5 year or more	16.0	64.0	9.0	36.0		14.0	56.0	11.0	44.0		16.0	64.0	9.0	36.0	
Work type	Full-time	110.0	67.5	53.0	32.5	0.66	95.0	58.3	68.0	41.7	0.61	107.0	65.6	56.0	34.4	0.91
	Part time	19.0	63.3	11.0	36.7		16.0	53.3	14.0	46.7		20.0	66.7	10.0	33.3	
Job satisfaction	Satisfied	78.0	59.5	53.0	40.5	0.00	69.0	52.7	62.0	47.3	0.05	78.0	59.5	53.0	40.5	0.01
	Unsatisfied	51.0	82.3	11.0	17.7		42.0	67.7	20.0	32.3		49.0	79.0	13.0	21.0	
Packed groceries items into bags after scanning	Yes	105.0	66.9	52.0	33.1	0.98	96.0	61.1	61.0	38.9	0.03	105.0	66.9	52.0	33.1	0.51
	No	24.0	66.7	12.0	33.3		15.0	41.7	21.0	58.3		22.0	61.1	14.0	38.9	
Received proper training to avoid WMSDs before starting work as a supermarket cashier	Yes	49.0	57.6	36.0	42.4	0.02	48.0	56.5	37.0	43.5	0.80	45.0	52.9	40.0	47.1	0.00
	No	80.0	74.1	28.0	25.9		63.0	58.3	45.0	41.7		82.0	75.9	26.0	24.1	
Number of workers at the same supermarket where the cashier works	>50 person	49.0	62.0	30.0	38.0	0.33	43.0	54.4	36.0	45.6	0.47	48.0	60.8	31.0	39.2	0.22
	<50 person	80.0	70.2	34.0	29.8		68.0	59.6	46.0	40.4		79.0	69.3	35.0	30.7	
Age (years), mean (SD)		27.2	6.4	27.2	6.6	0.91	27.6	50.9	26.6	49.1	0.27	27.0	49.4	27.6	50.6	0.37
BMI (kg/m^2^), mean (SD)		27.1	7.7	27.7	8.3	0.69	27.0	49.5	27.6	50.5	0.89	27.9	51.6	26.2	48.4	0.25
Number of breaks during a work shift, mean (SD)		1.7	1.1	1.7	1.3	0.54	1.6	47.8	1.8	52.2	0.48	1.6	44.3	2.0	55.7	0.04

**Table A2:** Upper limb.

		Shoulder					Elbow					Hand wrist				
		MSD Symptoms														
Variables		Yes		No		*p*	Yes		No		*p*	Yes		No		*p*
		N	%	N	N		N	N	%	N		N	N	%	N	
Gender	Male	73.0	52.1	67.0	47.9	0.13	30.0	21.4	110.0	78.6	0.12	53.0	37.9	87.0	62.1	0.24
	Female	34.0	64.2	19.0	35.8		17.0	32.1	36.0	67.9		25.0	47.2	28.0	52.8	
Marital status	Single	71.0	57.7	52.0	42.3	0.42	31.0	25.2	92.0	74.8	0.59	52.0	42.3	71.0	57.7	0.24
	Married	30.0	54.5	25.0	45.5		14.0	25.5	41.0	74.5		23.0	41.8	32.0	58.2	
	OTHER	6.0	40.0	9.0	60.0		2.0	13.3	13.0	86.7		3.0	20.0	12.0	80.0	
Dominant hand	right	95.0	55.9	75.0	44.1	0.74	42.0	24.7	128.0	75.3	0.76	69.0	40.6	101.0	59.4	0.89
	Left	12.0	52.2	11.0	47.8		5.0	21.7	18.0	78.3		9.0	39.1	14.0	60.9	
Educational level	Intermediate or less	12.0	52.2	11.0	47.8	0.25	6.0	26.1	17.0	73.9	0.56	7.0	30.4	16.0	69.6	0.42
	Secondary	64.0	52.0	59.0	48.0		27.0	22.0	96.0	78.0		50.0	40.7	73.0	59.3	
	Diploma	31.0	66.0	16.0	34.0		14.0	29.8	33.0	70.2		21.0	44.7	26.0	55.3	
Smoking status	Smoker	41.0	62.1	25.0	37.9	0.35	20.0	30.3	46.0	69.7	0.38	33.0	50.0	33.0	50.0	0.14
	Non smoker	60.0	51.3	57.0	48.7		25.0	21.4	92.0	78.6		41.0	35.0	76.0	65.0	
	X—smoker	6.0	60.0	4.0	40.0		2.0	20.0	8.0	80.0		4.0	40.0	6.0	60.0	
Practice sports times/week	No practice of sports	58.0	55.8	46.0	44.2	0.02	23.0	22.1	81.0	77.9	0.46	40.0	38.5	64.0	61.5	0.11
	1–3 times/week	43.0	63.2	25.0	36.8		20.0	29.4	48.0	70.6		33.0	48.5	35.0	51.5	
	4 times or more/week	6.0	28.6	15.0	71.4		4.0	19.0	17.0	81.0		5.0	23.8	16.0	76.2	
Using medications (any medication)	Yes	28.0	77.8	8.0	22.2	0.00	17.0	47.2	19.0	52.8	<0.001	23.0	63.9	13.0	36.1	0.00
	No	79.0	50.3	78.0	49.7		30.0	19.1	127.0	80.9		55.0	35.0	102.0	65.0	
Job experience (years)	≤one year	41.0	47.1	46.0	52.9	0.08	21.0	24.1	66.0	75.9	0.09	31.0	35.6	56.0	64.4	0.17
	2–4 year	52.0	64.2	29.0	35.8		24.0	29.6	57.0	70.4		39.0	48.1	42.0	51.9	
	5 year or more	14.0	56.0	11.0	44.0		2.0	8.0	23.0	92.0		8.0	32.0	17.0	68.0	
Work type	Full-time	91.0	55.8	72.0	44.2	0.80	38.0	23.3	125.0	76.7	0.43	64.0	39.3	99.0	60.7	0.45
	Part time	16.0	53.3	14.0	46.7		9.0	30.0	21.0	70.0		14.0	46.7	16.0	53.3	
Job satisfaction	Satisfied	64.0	48.9	67.0	51.1	0.01	25.0	19.1	106.0	80.9	0.01	47.0	35.9	84.0	64.1	0.06
	Unsatisfied	43.0	69.4	19.0	30.6		22.0	35.5	40.0	64.5		31.0	50.0	31.0	50.0	
Packed groceries items into bags after scanning	Yes	90.0	57.3	67.0	42.7	0.27	40.0	25.5	117.0	74.5	0.45	66.0	42.0	91.0	58.0	0.34
	No	17.0	47.2	19.0	52.8		7.0	19.4	29.0	80.6		12.0	33.3	24.0	66.7	
Received proper training to avoid WMSDs before starting work as a supermarket cashier	Yes	41.0	48.2	44.0	51.8	0.07	18.0	21.2	67.0	78.8	0.36	26.0	30.6	59.0	69.4	0.01
	No	66.0	61.1	42.0	38.9		29.0	26.9	79.0	73.1		52.0	48.1	56.0	51.9	
Number of workers at the same supermarket where the cashier works	>50 person	43.0	54.4	36.0	45.6	0.81	16.0	20.3	63.0	79.7	0.27	29.0	36.7	50.0	63.3	0.38
	<50 person	64.0	56.1	50.0	43.9		31.0	27.2	83.0	72.8		49.0	43.0	65.0	57.0	
Age (years), mean (SD)		27.4	50.5	26.9	49.5	0.43	27.5	50.4	27.1	49.6	0.55	27.3	50.1	27.1	49.9	0.85
BMI (kg/m^2^), mean (SD)		26.4	48.1	28.5	51.9	0.09	26.8	49.4	27.5	50.6	0.83	26.9	49.4	27.6	50.6	0.96
Number of breaks during a work shift, mean (SD)		1.6	46.8	1.8	53.2	0.40	1.5	45.2	1.8	54.8	0.17	1.5	44.9	1.8	55.1	0.04

**Table A3:** Lower limb.

		Hip					Knee					Ankle				
		MSD Symptoms														
Variables		Yes		No		*p*	Yes		No		*p*	Yes		No		*p*
		N	%	N	%		N	%	N	%		N	%	N	%	
Gender	Male	45.0	32.1	95.0	67.9	0.81	54.0	38.6	86.0	61.4	0.40	55.0	39.3	85.0	60.7	0.84
	Female	18.0	34.0	35.0	66.0		17.0	32.1	36.0	67.9		20.0	37.7	33.0	62.3	
Marital status	Single	38.0	30.9	85.0	69.1	0.27	44.0	35.8	79.0	64.2	0.52	48.0	39.0	75.0	61.0	0.89
	Married	22.0	40.0	33.0	60.0		23.0	41.8	32.0	58.2		22.0	40.0	33.0	60.0	
	OTHER	3.0	20.0	12.0	80.0		4.0	26.7	11.0	73.3		5.0	33.3	10.0	66.7	
Dominant hand	right	51.0	30.0	119.0	70.0	0.03	66.0	38.8	104.0	61.2	0.11	68.0	40.0	102.0	60.0	0.38
	Left	12.0	52.2	11.0	47.8		5.0	21.7	18.0	78.3		7.0	30.4	16.0	69.6	
Educational level	Intermediate or less	8.0	34.8	15.0	65.2	0.97	10.0	43.5	13.0	56.5	0.07	7.0	30.4	16.0	69.6	0.62
	Secondary	40.0	32.5	83.0	67.5		38.0	30.9	85.0	69.1		48.0	39.0	75.0	61.0	
	Diploma	15.0	31.9	32.0	68.1		23.0	48.9	24.0	51.1		20.0	42.6	27.0	57.4	
Smoking status	Smoker	26.0	39.4	40.0	60.6	0.26	23.0	34.8	43.0	65.2	0.91	33.0	50.0	33.0	50.0	0.01
	Non smoker	33.0	28.2	84.0	71.8		44.0	37.6	73.0	62.4		36.0	30.8	81.0	69.2	
	X—smoker	4.0	40.0	6.0	60.0		4.0	40.0	6.0	60.0		6.0	60.0	4.0	40.0	
Practice sports times/week	No practice of sports	39.0	37.5	65.0	62.5	0.20	40.0	38.5	64.0	61.5	0.82	42.0	40.4	62.0	59.6	0.14
	1–3 times/week	20.0	29.4	48.0	70.6		23.0	33.8	45.0	66.2		29.0	42.6	39.0	57.4	
	4 times or more/week	4.0	19.0	17.0	81.0		8.0	38.1	13.0	61.9		4.0	19.0	17.0	81.0	
Using medications (any medication)	Yes	20.0	55.6	16.0	44.4	0.00	18.0	50.0	18.0	50.0	0.07	21.0	58.3	15.0	41.7	0.01
	No	43.0	27.4	114.0	72.6		53.0	33.8	104.0	66.2		54.0	34.4	103.0	65.6	
Job experience (years)	≤one year	25.0	28.7	62.0	71.3	0.20	23.0	26.4	64.0	73.6	0.02	31.0	35.6	56.0	64.4	0.57
	2–4 year	32.0	39.5	49.0	60.5		38.0	46.9	43.0	53.1		35.0	43.2	46.0	56.8	
	5 year or more	6.0	24.0	19.0	76.0		10.0	40.0	15.0	60.0		9.0	36.0	16.0	64.0	
Work type	Full-time	52.0	31.9	111.0	68.1	0.61	55.0	33.7	108.0	66.3	0.04	64.0	39.3	99.0	60.7	0.79
	Part time	11.0	36.7	19.0	63.3		16.0	53.3	14.0	46.7		11.0	36.7	19.0	63.3	
Job satisfaction	Satisfied	39.0	29.8	92.0	70.2	0.22	41.0	31.3	90.0	68.7	0.02	46.0	35.1	85.0	64.9	0.12
	Unsatisfied	24.0	38.7	38.0	61.3		30.0	48.4	32.0	51.6		29.0	46.8	33.0	53.2	
Packed groceries items into bags after scanning	Yes	52.0	33.1	105.0	66.9	0.77	60.0	38.2	97.0	61.8	0.39	68.0	43.3	89.0	56.7	0.01
	No	11.0	30.6	25.0	69.4		11.0	30.6	25.0	69.4		7.0	19.4	29.0	80.6	
Received proper training to avoid WMSDs before starting work as a supermarket cashier	Yes	26.0	30.6	59.0	69.4	0.59	20.0	23.5	65.0	76.5	0.00	24.0	28.2	61.0	71.8	0.01
	No	37.0	34.3	71.0	65.7		51.0	47.2	57.0	52.8		51.0	47.2	57.0	52.8	
Number of workers at the same supermarket where the cashier works	>50 person	24.0	30.4	55.0	69.6	0.58	25.0	31.6	54.0	68.4	0.22	27.0	34.2	52.0	65.8	0.27
	<50 person	39.0	34.2	75.0	65.8		46.0	40.4	68.0	59.6		48.0	42.1	66.0	57.9	
Age (years), mean (SD)		26.9	49.6	27.3	50.4	0.67	27.5	50.5	27.0	49.5	0.59	27.3	50.2	27.1	49.8	0.87
BMI (kg/m^2^), mean (SD)		27.4	50.1	27.3	49.9	0.99	28.4	51.5	26.7	48.5	0.05	27.7	50.6	27.1	49.4	0.40
Number of breaks during a work shift, mean (SD)		1.7	49.9	1.7	50.1	0.77	1.5	45.0	1.8	55.0	0.04	1.5	43.8	1.9	56.2	0.02
